# Neuroaesthetics: exploring the role of aesthetic experience in neurorehabilitation

**DOI:** 10.3389/fpsyg.2025.1671220

**Published:** 2025-12-03

**Authors:** Francesca Colombi, Greta Varesio, Enrico Selini, Elia Amighetti, Maura Crepaldi, Giulia Fusi, Irene Ronga, Alice Cancer, Alessandro Antonietti, Giuliano Carlo Geminiani, Maria Luisa Rusconi

**Affiliations:** 1 Department of Human and Social Sciences, University of Bergamo, Bergamo, Italy; 2 Department of Psychology, University of Torino, Turin, Italy; 3 Department of Psychology, Catholic University of the Sacred Heart, Milan, Italy

**Keywords:** neuroaesthetics, cognitive rehabilitation, aesthetic appreciation, emotional regulation, art-based interventions, brain plasticity, preferred music, neurorehabilitation

## Abstract

The rise in life expectancy, along with other factors, leads to an increase in neurodegenerative diseases and stroke. Research in neuropsychological fields is working toward developing innovative methods to address and treat the cognitive disorders that can result from these conditions, such as non-pharmacological interventions (e.g., cognitive stimulation training). As an emerging field, neuroaesthetics explores the cognitive processes and neural foundations associated with aesthetic experiences. As demonstrated by the “Michelangelo Effect,” observing artworks stimulates brain regions and emotional responses that could positively impact the effectiveness of neurorehabilitation protocols. This article aims to present an overview of existing theories and models that provide a framework for understanding the possible role of aesthetic appreciation in neurological and cognitive rehabilitation.

## 1 Introduction

The “sublime,” according to Kant (1790), is an aesthetic experience capable of activating cognitive faculties and arousing deep emotional involvement. Contemporary neuroscience confirms this intuition, showing that exposure to visual art and music can activate neural circuits linked to pleasure and motivation, and even promote brain plasticity. These findings open new perspectives for neurorehabilitation, suggesting that aesthetic stimuli cannot only enrich the patient’s experience, but also actively modulate functional recovery.

Neuroaesthetics is a relatively new field of research that seeks to understand the neural substrates of human aesthetic experiences (Mastandrea et al., 2019). The aesthetic experience is a multifaceted phenomenon and emerges from the interaction of three neural systems: sensory–motor, emotion–valuation, meaning–knowledge, forming the aesthetic triad (Brown et al., 2011; Chatterjee and Vartanian, 2014). Aesthetic appreciation consists in liking and enjoying an object and involves perceptual, cognitive, emotional, and evaluative engagement (Jacobsen, 2006; Leder et al., 2004). Findings from empirical aesthetics and neuroimaging studies suggest that aesthetic appreciation emerges from the interaction between the basic features of the object and the dynamics of perceptual processing of the perceiver (Reber et al., 2004; Sarasso et al., 2020; Table 1).

**TABLE 1:** Glossary of terms.

Term	Definition
Neuroaesthetics	The study of the neural basis of human aesthetic appreciation, combining neuroscience and cognitive science.
Aesthetic experience	The experience that is directed to an object we can appraise and involves different neural systems: sensory–motor, emotion–valuation, and meaning–knowledge.
Aesthetic appreciation	The experience of liking an object or an experience, which can be influenced by different factors, such as subjective preferences, object’s features, contextual and/or other cultural factors.

Neuroaesthetics involves the analysis of human cognition and behavior through methods from cognitive neuroscience, bringing together the cognitive and neural levels of explanation (Pearce et al., 2016). In recent years the interest in neuroaesthetics has expanded, also with a focus on its potential application in neurorehabilitation. In this context, the integration of aesthetic stimuli into neurorehabilitation programs could represent an innovative strategy for enhancing functional recovery. For example, aesthetic appreciation has been linked with certain harmonic characteristics of the visual stimuli, leading to an ease of information processing that was recently associated with ecological affordance (De Bartolo et al., 2022), thereby suggesting a potential role in enhancing rehabilitative processes. Aesthetic stimuli, such as music and visual arts, can positively influence cognitive processes (see section 3) through the modulation of brain plasticity and foster patient involvement, potentially playing a significant role in neurorehabilitation (Oliva et al., 2023).

Music has been extensively studied for its impact on cognitive and motor functions (e.g., Dalla Bella, 2018; Sella et al., 2024) and its benefits include an increase in attentional engagement (Jenkins, 2001; Sarasso et al., 2022a). Similarly, artistic visual stimuli have shown significant potential in neurorehabilitation. The “Michelangelo Effect” describes a phenomenon in which improvements in motor performance are observed, following the observation of artistic stimuli (Iosa et al., 2021). This effect is attributed to the ability of artistic beauty to activate specific brain areas, including sensorimotor areas, promoting motor recovery and patient engagement (Di Dio et al., 2016; Freedberg and Gallese, 2007; Umiltá et al., 2012).

This perspective article explores the potential of neuroaesthetics within neurorehabilitation, analyzing the role of aesthetic experiences in modulating emotional responses and activating specific brain areas. Considering the increase in life expectancy and the consequent incidence of neurodegenerative diseases and stroke, this paper proposes a reflection on the possible applications of neuroaesthetics as an innovative and complementary approach to traditional strategies of cognitive rehabilitation. This perspective article aims to critically reconsider the role of aesthetic experience in neurorehabilitation, proposing that aesthetic gratification is not merely a secondary by-product but a central mechanism for enhancing engagement, motivation, and neuroplasticity.

## 2 Neural correlates of aesthetic experience

Aesthetic experience involves the activation of different brain areas, which vary depending on several factors, such as the type of stimulus and subjective preferences.

Several studies (Chib et al., 2013; Ferrari et al., 2015; Mas-Herrero et al., 2018) highlighted the activation of the prefrontal cortex while experiencing aesthetic stimuli, whose involvement is often related to the modulation of frontostriatal excitability and top-down attentional control. Prefrontal cortex stimulation is also directly linked to the activation of the Default Mode Network (DMN) during aesthetic experiences (Cela-Conde et al., 2013; Vessel et al., 2012). Kawabata and Zeki (2004) found increased activity in the medial orbitofrontal cortex (mOFC) in response to beautiful stimuli, while increased activation in the motor area was observed following the presentation of ugly stimuli (for a review and meta-analysis focused on the interplay between aesthetic appreciation and motor activation/inhibition, refer to Sarasso et al., 2020; Sacheli et al., 2022). Vartanian and Goel (2004) found that activity in the right caudate nucleus, in the left cingulate gyrus, and in the occipital gyri is positively correlated with the enjoyment of preferred aesthetic stimuli.

Aesthetic experience not only activates specific brain areas but also affects brain plasticity. Neuronal circuits that are activated by pleasure or positive rewards tend to become more stable and efficient over time. Pleasure and reward support the consolidation of neuronal circuits and promote the creation of new associations through the release of dopamine, which contributes to the construction of new excitatory synapses and enhances neural transmission (Canali, 2021).

Finally, neuroimaging evidence explains the feeling of satisfaction we experience when listening to music we enjoy. A causal relationship between the reward circuitry and the modulation of attributed hedonic value has been observed (Pecina et al., 2006; Peng et al., 2015), a result in line with clinical findings of a diminished connection between the auditory areas responsible for the representation of music and the reward network in patients with music anhedonia (Martınez-Molina et al., 2016; Sachs et al., 2016).

## 3 Underlying mechanism of artistic interventions in neurorehabilitation

The use of artistic interventions as a complement to traditional neurorehabilitation has gained increasing attention due to their potential to engage multiple sensorimotor, cognitive, and emotional processes. Various forms of artistic expression have been integrated into therapeutic protocols, demonstrating positive effects on patients with neurological and cognitive impairments (Iosa et al., 2021; Joschko et al., 2024; Ree et al., 2025; Valverde-Guijarro et al., 2022; Windle et al., 2018), as well as promoting emotional and psychological wellbeing (Beaudry et al., 2020; Liu et al., 2024; Raglio et al., 2015; Windle et al., 2018).

Art and aesthetically gratifying stimuli can be employed to stimulate specific rehabilitative mechanisms depending on the nature of the pathology and the goals of the intervention. For instance, language—in conditions such as non-fluent aphasia—as well as motor abilities can be rehabilitated through music-based interventions (Koshimori et al., 2025) and dance activities that emphasize rhythm and synchronization (Dalla Bella, 2018). Owing to the temporal regularity and predictability of music, such interventions facilitate bodily engagement and the coordination of movement with rhythmic auditory stimuli or with the movements of others, thereby guiding attentional processes (Dalla Bella, 2018; Cucca et al., 2021; Tommasini et al., 2022) and enhancing spatial navigation skills (Valverde-Guijarro et al., 2022) and speech abilities such as fluency and articulation (Gu et al., 2024; Koshimori et al., 2025). At the same time, visuospatial exploration and sensorimotor processing abilities can be rehabilitated through visual art activities and the use of innovative technological tools, such as virtual reality (VR), which promote the activation of brain regions involved in spatial exploration and the pragmatic encoding of movement (Iosa et al., 2021).

Such interventions actually share an aesthetic gratification component that is often overlooked in neurorehabilitation, where attention tends to focus on the intrinsic properties of artistic stimuli rather than on the mechanisms through which aesthetic experience and gratification enhance therapeutic outcomes. We argue that the component of aesthetic gratification, from a subjective perspective, may exert a positive influence on both cognitive and affective dimensions by enhancing factors such as pleasure, social engagement, and wellbeing (Lomas, 2016; Windle et al., 2018; Mastandrea et al., 2019; Beaudry et al., 2020). This dimension can therefore serve as a reinforcing factor within rehabilitative activities, providing added value compared to traditional interventions that lack an aesthetic component.

### 3.1 Cognitive pathways: attention, perception, and plasticity

Artistic interventions can enhance attentional and perceptual processes, contributing to improve rehabilitation outcomes. Recent studies are moving in the encouraging direction of examining effects of aesthetic appreciation on cognitive abilities. It has been observed that engaging with appreciated music or visual content can enhance perceptual learning, memorization, and attentional processes (Rolke et al., 2019; Sarasso et al., 2020, 2021, Sarasso et al., 2022a,b), suggesting that the most aesthetically appreciated stimuli can entail privileged cognitive processing pathways.

Other studies (Sarasso et al., 2022a,b) explored links between exposure to preferred auditory stimuli and electrophysiological indices of implicit learning. Enhanced Mismatch Negativity (MMN) responses indexing modulation of implicit learning processing were observed after listening to preferred musical pieces (Sarasso et al., 2022a) and during the presentation of preferred musical intervals (Sarasso et al., 2022b). The MMN response, enhanced for preferred stimuli, was found to be correlated with trial-by-trial fluctuation in Bayesian surprise (Sarasso et al., 2022a,b), indicating that the precision weighting in Bayesian perceptual learning is related to aesthetic appreciation. Additionally, during preferred music listening, an enhancement in alpha power was observed, which was suggested to index cognitive enhancement (Sarasso et al., 2022a).

Exposure to preferred auditory stimuli is linked to reduced interoceptive awareness, indexed by lower heartbeat detection accuracy, suggesting an attentional shift from self to environment, which may explain enhanced perceptual learning (Sarasso et al., 2022a). Another study found enhanced memorization performance of preferred musical chords, compared to non-preferred ones, reflecting a relationship between aesthetic appreciation and strengthened higher-order processing (Sarasso et al., 2021). Similar results were found in the visual domain: ERPs indexing attentional processes and related alpha desynchronization were stronger for appreciated visual stimuli (Sarasso et al., 2020). In addition, aesthetic appreciation has also been associated with the process of insight, proposing that the “Aha! moment” can be linked to the acquisition of awareness on the result of complex cognitive processes. In this insight process, the activity of the DMN can play a role (Cela-Conde et al., 2013). Studies showed that insight and its intensity predict subsequent aesthetic appreciation (Muth and Carbon, 2013), highlighting reflective and active processing that elicits pleasure in the cognitive process of insight.

### 3.2 Affective pathways: emotional regulation, and processing

Aesthetic experiences play a crucial role in emotional regulation and affective wellbeing. The cognitive processing of artistic objects gives rise to affective experiences, often positive, which contribute to emotional balance and psychological health (Mastandrea et al., 2019). According to the staged model devised by Leder et al. (2004), aesthetic pleasure depends on the understanding of the artwork: The greater the understanding and the lower the ambiguity, the more likely it is to elicit positive affective experiences that contribute to emotional regulation. Neurophysiological findings support this view, showing that contextual factors promoting comprehension increase activity in regions such as mOFC and ventromedial prefrontal cortex, areas associated with reward and emotional processing (Mastandrea et al., 2019).

Chatterjee and Vartanian (2014) emphasized that emotions lie at the core of aesthetic experience. Artistic stimuli engage brain regions related to empathy and interoception, including the bilateral insula, and activate the default mode network (DMN), fostering internal reflection and deep emotional processing. This suggests that art can modulate and regulate emotional states, promoting deep emotional processing.

Art can also transform negative emotions into pleasurable experiences. Several theories explained how art evokes paradoxical pleasure, even from negative emotions (Sachs et al., 2015). The concept of “psychological distance” reduces the direct emotional impact, facilitating aesthetic appreciation of negative content. The perception of safety in artistic appreciation allows emotions such as sadness and pain to be experienced, turning them into pleasure. Empathic responses to art are mediated by meta-emotional appreciation (Mastandrea et al., 2019). In music, the BRECVEMA model proposed by Juslin (2013) explains how emotional contagion and aesthetic judgment allow listeners to experience and regulate emotions simultaneously. Listening to sad music, for example, can restore emotional balance by engaging reward- and homeostasis-related brain areas such as the orbitofrontal cortex, nucleus accumbens, insula, and cingulate cortex (Sachs et al., 2015).

Czepiel et al. (2023) also explored the physiological measures elicited during ecological music listening, showing enhanced involuntary and voluntary measures respectively indexing the sensory and the liking aspects linked to the aesthetic experience. Crucially, they found an enhancement in the self-report measures of aesthetic experiences during the multimodal listening compared to the audio-only condition (Czepiel et al., 2023).

Similarly, aesthetic pleasure from visual arts depends on: (1) emotional contagion; (2) evaluation of emotional valence; (3) regulating emotions accordingly; (4) enjoying the aesthetic experience and formulating an aesthetic judgment. An aesthetically pleasurable experience can be considered rewarding. These processes find a common neural substrate in emotional processing circuits and reward networks, suggesting that the integration of neuroimaging and physiological measurements could clarify the link between aesthetic experience and wellbeing (Mastandrea et al., 2019).

## 4 Discussion

In addition to the growing interest in non-pharmacological therapeutic approaches, such as VR (D’Cunha et al., 2019; Macchitella et al., 2023) and mindfulness (Han, 2022), we propose an approach with a specific focus on aesthetic gratification that we believe it could offer significant potential for enhancing patient-centered approaches in cognitive rehabilitation, enhancing cognitive and motivational dimensions respectively through neural plasticity and the dopaminergic system (see section 2). Unlike VR or mindfulness, which represent structured interventions requiring specific training or equipment, the aesthetic experience does not constitute a set of exercises but rather a condition that precedes or accompanies rehabilitation. It can therefore be integrated into therapeutic contexts without the constraints of environment, specialized personnel, or technology. Moreover, while mindfulness promotes a distinct mental state (e.g., non-judgmental awareness) and VR relies on immersive simulation that may not be suitable for all patients (e.g., cybersickness), neuroaesthetic stimulation is low-cost, easily applicable, and inherently adaptable, relying on the presentation of visual or auditory stimuli. The evidence reviewed in the previous sections points to a direct link between exposure to aesthetically pleasing stimuli and the enhancement of perceptual and learning processes. To our knowledge, however, techniques such as VR and mindfulness do not automatically produce such perceptual/learning effects. In other words, the contribution of aesthetic gratification does not appear to be merely motivational. Rather, moments of aesthetic experience may play a crucial role in systematically increasing attentional engagement with external stimuli (Barbieri et al., 2025) and in enhancing perceptual and learning processes (Rolke et al., 2019; Sarasso et al., 2020, 2021, Sarasso et al., 2022a,b). These findings suggest that, besides classically investigated motivational effects, aesthetic pleasure seems to act as a true cognitive amplifier, fostering a deeper, more efficient interaction between the individual and their environment. Furthermore, it is important to note that incorporating an aesthetic element into rehabilitative treatments does not require specialized expertise from the practitioner, nor does it prolong or complicate the sessions. It therefore represents a sustainable opportunity within the broader context of rehabilitation.

Research has already highlighted the potential of aesthetic experiences in modulating emotional and cognitive responses but much remains to be done to translate this knowledge into structured and replicable protocols and capitalize on this under-explored opportunity. Despite the great potential of aesthetics-based approaches, there is an important gap in literature: To our knowledge, there are indeed only a few studies on standardized protocols for the integration of neuroaesthetics in cognitive rehabilitation. As mentioned before, some studies investigated the benefits of aesthetic appreciation on cognition (for reference, see section 3), but the methodologies for systematically integrating these elements into neurorehabilitation pathways remain poorly defined. This lack of standardization is an obstacle to the widespread adoption of neuroaesthetics in clinical practice. To overcome this critical issue, interdisciplinary collaboration between neuroscientists, psychologists, and clinicians will be essential. Rigorous studies and evidence-based guidelines are needed to demonstrate the effectiveness of aesthetic stimulation in neurorehabilitation.

As recently reported by Bokoch et al. (2025), within the field of art therapy, the variability of interventions makes the process of comparing different studies more complex when assessing their effectiveness. The authors therefore advocate the use of multiple qualitative and quantitative measures to strengthen the evaluation of intervention outcomes, which appear promising, particularly from a neuroscientific perspective.

A key aspect for the future will be the development and validation of protocols based on the principles of neuroaesthetics that can enhance neurorehabilitation interventions. This means identifying the most effective forms of aesthetic stimulation (e.g., auditory or visual stimuli) and understanding the specific neural mechanisms by which these experiences influence cognitive function. Furthermore, longitudinal studies will be essential to assess the long-term effects of such interventions in patients with neurodegenerative diseases or disorders resulting from brain injury.

For example, one area in which these approaches could be particularly useful is the cognitive rehabilitation of patients with Mild Cognitive Impairment (MCI), a transitional condition between healthy aging and dementia, which is considered a “window” for interventions aimed at slowing disease progression (Anderson, 2019). Aesthetic stimulation could enhance residual abilities and, when integrated into cognitive training, promote both the maintenance of cognitive functions and increase the level of motivation and emotional engagement, key elements to ensure the effectiveness of cognitive stimulation protocols.

**FIGURE 1:**
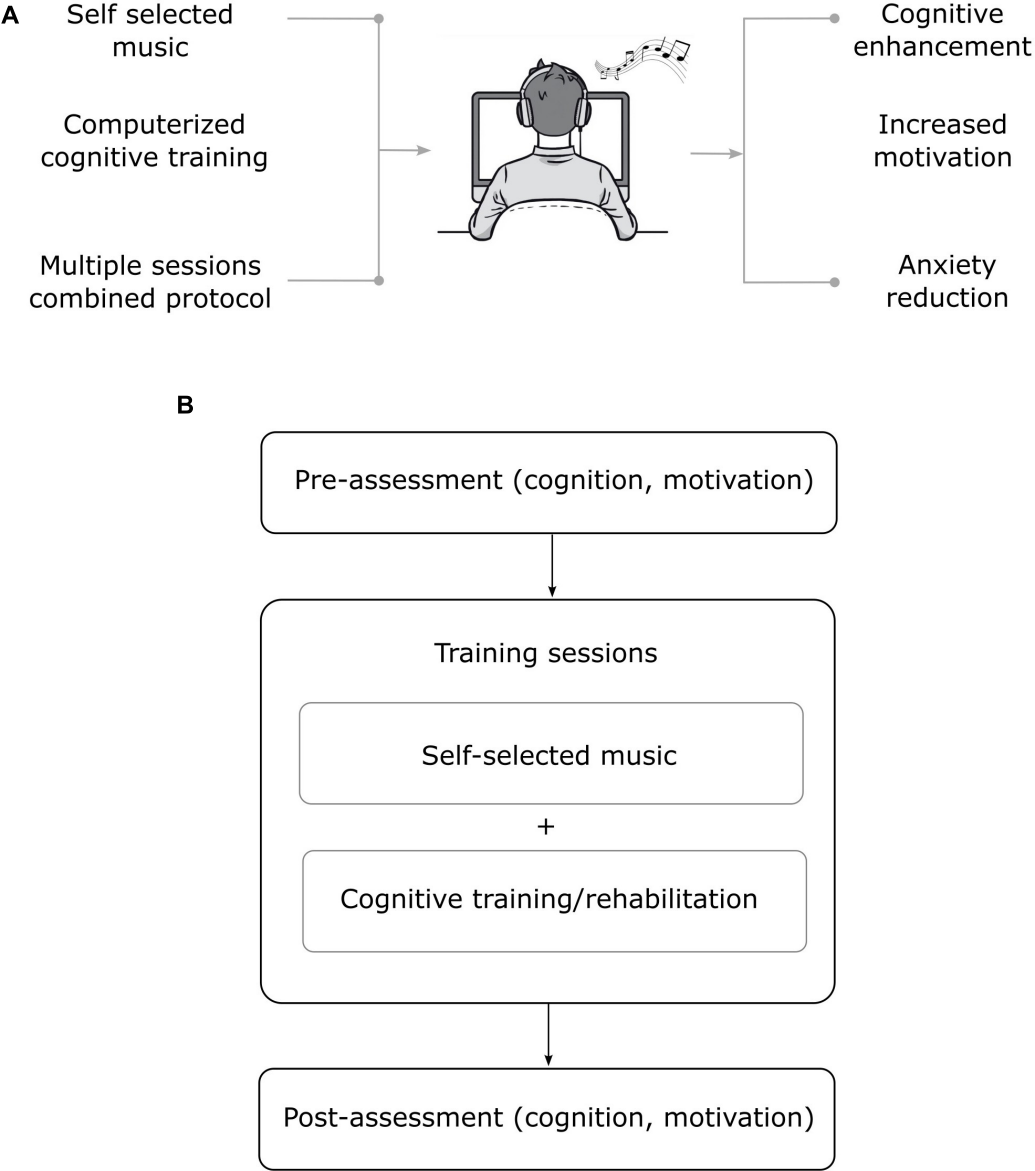
**(A)** Different aspects of the proposed protocols (self-selected music; computerized cognitive training; multiple sessions combined protocol) and different outcomes (cognitive enhancement; increased motivation; anxiety reduction). **(B)** Flowchart illustrating the proposed neuroaesthetic-rehabilitation protocol. The protocol includes a pre-assessment phase (evaluation of cognition and motivation), followed by multiple training sessions integrating self-selected music with computerized cognitive training/rehabilitation. A post-assessment phase (cognition and motivation) concludes the intervention.

A particularly interesting aspect concerns the use of aesthetics as an integration into traditional neuropsychological therapies. We hypothesize that customizing these interventions according to individual preferences could further increase their effectiveness, making them not only more functional, but also more rewarding (Sarasso et al., 2022a,b). Such customization may also influence psychological dimensions: For example, preferred music listening has been associated with reduced anxiety (Barbieri et al., 2024). A protocol proposal might be to listen to favorite songs or look at favorite works of art before starting cognitive training, assessing whether this artistic exposure could increase the effectiveness of the training. This approach could foster greater emotional and cognitive activation, improving patients’ performance in rehabilitation activities, while requiring no specific training for the rehabilitator and allowing for easy integration into traditional interventions without additional resources (Figure 1A).

In this perspective, we aim to propose a neurorehabilitation protocol that would integrate aesthetic stimulation into cognitive training sessions. The intervention should enhance emotional engagement and cognitive performance through exposure to aesthetic stimuli such as self-selected music. In practical terms, the proposed intervention would consist of a 10-session individual online training program administered to the patients.

Participants in the experimental group would receive a neuroaesthetic module—comprising several pieces of music chosen by the patients themselves (see section 3.1)—administered for 10–15 min before a 30-min cognitive training session. Cognitive training includes exercises that can be adapted to the different clinical needs of patients, for example those with MCI or stroke outcomes, ranging from rehabilitation to cognitive stimulation. The expected outcome is an improvement in cognitive functions and treatment motivation. These aspects would be assessed before and after the training sessions (Figure 1B).

Since the proposed protocol represents an innovation within the field of cognitive enhancement techniques, an initial assessment of cognitive and psychological outcomes using behavioral measures, including self-report questionnaires, would allow for a preliminary evaluation of their effectiveness. Consequently, integrating these findings with neuroimaging techniques will be useful to better assess the neural correlates associated with the proposed intervention. Furthermore, the use of neuroimaging tools and neurophysiological measures could provide new insights into the neural mechanisms involved, paving the way for more targeted and personalized strategies.

In conclusion, neuroaesthetics might have the potential to transform neurorehabilitation by offering innovative, engaging, and cognitively stimulating experiences. Nevertheless, in order for this potential to be produced and translated into concrete benefits for patients, further research is required.

## References

[B1] Anderson N. D. (2019). State of the science on mild cognitive impairment (MCI). *CNS Spectr.* 24 78–87. 10.1017/S1092852918001347 30651152

[B2] Barbieri P. Berto M. Sarasso P. Frascaroli J. Handjaras G. Piovesan F. (2025). Unveiling the relationship between aesthetic experiences and attention through a cross-experiment validation of their processing biomarkers. *PNAS Nexus* 4:gaf288. 10.1093/pnasnexus/pgaf288 41064242 PMC12501847

[B3] Barbieri P. Sarasso P. Lodico F. Aliverti A. Murayama K. Sacco K. (2024). The aesthetic valve: How aesthetic appreciation may switch emotional states from anxiety to curiosity. *Philos. Trans. R. Soc. Lond. B Biol. Sci.* 379:20220413. 10.1098/rstb.2022.0413 38104608 PMC10725764

[B4] Beaudry L. Fortin S. Rochette A. (2020). Adapted dance used in subacute rehabilitation post-stroke: Impacts perceived by patients, relatives and rehabilitation therapists: Qualitative study. *Disabil. Rehabil.* 42 2997–3006. 10.1080/09638288.2019.1581845 30907140

[B5] Bokoch R. Hass-Cohen N. Espinoza A. O’Reilly T. Levi E. (2025). A scoping review of integrated arts therapies and neuroscience research. *Front. Psychol.* 16:1569609. 10.3389/fpsyg.2025.1569609 40406613 PMC12095372

[B6] Brown S. Gao X. Tisdelle L. Eickhoff S. B. Liotti M. (2011). Naturalizing aesthetics: Brain areas for aesthetic appraisal across sensory modalities. *Neuroimage* 58 250–258. 10.1016/j.neuroimage.2011.06.012 21699987 PMC8005853

[B7] Canali S. (2021). *Regolare le emozioni. Teorie e metodi per lo sviluppo e il potenziamento dell’autocontrollo.* Roma: Carocci Editore.

[B8] Cela-Conde C. J. García-Prieto J. Ramasco J. J. Mirasso C. R. Bajo R. Munar E. (2013). Dynamics of brain networks in the aesthetic appreciation. *Proc. Natl. Acad. Sci. U. S. A.* 110 (Suppl. 2), 10454–10461. 10.1073/pnas.1302855110 23754437 PMC3690613

[B9] Chatterjee A. Vartanian O. (2014). Neuroaesthetics. *Trends Cogn. Sci.* 18 370–375. 10.1016/j.tics.2014.03.003 24768244

[B10] Chib V. S. Yun K. Takahashi H. Shimojo S. (2013). Noninvasive remote activation of the ventral midbrain by transcranial direct current stimulation of prefrontal cortex. *Transl. Psychiatry* 3:e268. 10.1038/tp.2013.44 23756377 PMC3693403

[B11] Cucca A. Di Rocco A. Acosta I. Beheshti M. Berberian M. Bertisch H. C. (2021). Art therapy for Parkinson’s disease. *Parkinson. Relat. Disord.* 84 148–154. 10.1016/j.parkreldis.2021.01.013 33526323

[B12] Czepiel A. Fink L. K. Seibert C. Scharinger M. Kotz S. A. (2023). Aesthetic and physiological effects of naturalistic multimodal music listening. *Cognition* 239:105537. 10.1016/j.cognition.2023.105537 37487303

[B13] Dalla Bella S. (2018). Music and movement: Towards a translational approach. *Neurophysiol. Clin.* 48 377–386. 10.1016/j.neucli.2018.10.067 30396753

[B14] D’Cunha N. M. Nguyen D. Naumovski N. McKune A. J. Kellet J. Georgousopoulou E. N. (2019). A mini-review of virtual reality-based interventions to promote well-being for people living with dementia and mild cognitive impairment. *Gerontology* 65 430–440. 10.1159/000500040 31108489

[B15] De Bartolo D. De Luca M. Antonucci G. Schuster S. Morone G. Paolucci S. (2022). The golden ratio as an ecological affordance leading to aesthetic attractiveness. *PsyCh J.* 11 729–740. 10.1002/pchj.505 34951139 PMC9787369

[B16] Di Dio C. Ardizzi M. Massaro D. Di Cesare G. Gilli G. Marchetti A. (2016). Human, nature, dynamism: The effects of content and movement perception on brain activations during the aesthetic judgment of representational paintings. *Front. Hum. Neurosci.* 9:705. 10.3389/fnhum.2015.00705 26793087 PMC4709505

[B17] Ferrari C. Lega C. Tamietto M. Nadal M. Cattaneo Z. (2015). I find you more attractive after (prefrontal cortex) stimulation. *Neuropsychologia* 72 87–93. 10.1016/j.neuropsychologia.2015.04.024 25912761

[B18] Freedberg D. Gallese V. (2007). Motion, emotion and empathy in esthetic experience. *Trends Cogn. Sci.* 11 197–203. 10.1016/j.tics.2007.02.003 17347026

[B19] Gu J. Long W. Zeng S. Li C. Fang C. Zhang X. (2024). Neurologic music therapy for non-fluent aphasia: A systematic review and meta-analysis of randomized controlled trials. *Front. Neurol.* 15:1395312. 10.3389/fneur.2024.1395312 38846040 PMC11153767

[B20] Han A. (2022). Mindfulness-Based interventions for older adults with dementia or mild cognitive impairment: A meta-analysis. *Clin. Gerontol.* 45 763–776. 10.1080/07317115.2021.1995561 34693892

[B21] Iosa M. Aydin M. Candelise C. Coda N. Morone G. Antonucci G. (2021). The michelangelo effect: Art improves the performance in a virtual reality task developed for upper limb neurorehabilitation. *Front. Psychol.* 11:611956. 10.3389/fpsyg.2020.611956 33488478 PMC7817887

[B22] Jacobsen T. (2006). Bridging the arts and sciences: A framework for the psychology of aesthetics. *Leonardo* 39 155–162. 10.1162/leon.2006.39.2.155

[B23] Jenkins J. S. (2001). The Mozart effect. *J. R. Soc. Med.* 94 170–172. 10.1177/014107680109400404 11317617 PMC1281386

[B24] Joschko R. Klatte C. Grabowska W. A. Roll S. Berghöfer A. Willich S. N. (2024). Active visual art therapy and health outcomes: A systematic review and meta-analysis. *JAMA Netw. Open* 7:e2428709. 10.1001/jamanetworkopen.2024.28709 39264631 PMC11393726

[B25] Juslin P. N. (2013). From everyday emotions to aesthetic emotions: Towards a unified theory of musical emotions. *Phys. Life Rev.* 10 235–266. 10.1016/j.plrev.2013.05.008 23769678

[B26] Kant I. (1790). *The critique of judgment*. ed. Pluhar W. S. (Indianapolis, IN: Hackett) 1987.

[B27] Kawabata H. Zeki S. (2004). Neural correlates of beauty. *J. Neurophysiol.* 91 1699–1705. 10.1152/jn.00696.2003 15010496

[B28] Koshimori Y. Akkunje P. S. Tjiandri E. Kowaleski J. B. Thaut M. H. (2025). Music-based interventions for nonfluent aphasia: A systematic review of randomized control trials. *Ann. N. Y. Acad. Sci.* 1549 92–111. 10.1111/nyas.15387 40543062 PMC12309435

[B29] Leder H. Belke B. Oeberst A. Augustin D. (2004). A model of aesthetic appreciation and aesthetic judgments. *Br. J. Psychol.* 95 489–508. 10.1348/0007126042369811 15527534

[B30] Liu S. Huang X. Liu Y. Yue J. Li Y. Chen L. (2024). A scoping review of the use of creative activities in stroke rehabilitation. *Clin. Rehabil.* 38 497–509. 10.1177/02692155241227049 38232974

[B31] Lomas T. (2016). Positive art: Artistic expression and appreciation as an exemplary vehicle for flourishing. *Rev. Gen. Psychol.* 20 171–182. 10.1037/gpr0000073

[B32] Macchitella L. Amendola S. Barraco G. Scoditti S. Gallo I. Oliva M. C. (2023). A narrative review of the use of a cutting-edge vitation. *NeuroRehabilitation* 53 439–457. 10.3233/NRE-230066 38143388 PMC10789333

[B33] Martınez-Molina N. Mas-Herrero E. Rodrıguez-Fornells A. Zatorre R. J. MarcoPallares J. (2016). Neural correlates of specific musical anhedonia. *Proc. Natl. Acad. Sci. U. S. A.* 113 E7337–E7345. 10.1073/pnas.1611211113 27799544 PMC5135354

[B34] Mas-Herrero E. Dagher A. Zatorre R. J. (2018). Modulating musical reward sensitivity up and down with transcranial magnetic stimulation. *Nat. Hum. Behav.* 2 27–32. 10.1038/s41562-017-0241-z 30980048

[B35] Mastandrea S. Fagioli S. Biasi V. (2019). Art and psychological well-being: Linking the brain to the aesthetic emotion. *Front. Psychol.* 10:739. 10.3389/fpsyg.2019.00739 31019480 PMC6458291

[B36] Muth C. Carbon C. C. (2013). The aesthetic aha: On the pleasure of having insights into gestalt. *Acta Psychol.* 144 25–30. 10.1016/j.actpsy.2013.05.001 23743342

[B37] Oliva A. Iosa M. Antonucci G. De Bartolo D. (2023). Are neuroaesthetic principles applied in art therapy protocols for neurorehabilitation? A systematic mini-review. *Front. Psychol.* 14:1158304. 10.3389/fpsyg.2023.1158304 37377696 PMC10291050

[B38] Pearce M. T. Zaidel D. W. Vartanian O. Skov M. Leder H. Chatterjee A. (2016). Neuroaesthetics: The cognitive neuroscience of aesthetic experience. *Perspect. Psychol. Sci.* 11 265–279. 10.1177/1745691615621274 26993278

[B39] Pecina S. Smith K. S. Berridge K. C. (2006). Hedonic hot spots in the brain. *Neuroscientist* 12 500–511. 10.1177/1073858406293154 17079516

[B40] Peng Y. Gillis-Smith S. Jin H. Tränkner D. Ryba N. J. Zuker C. S. (2015). Sweet and bitter taste in the brain of awake behaving animals. *Nature* 527 512–515. 10.1038/nature15763 26580015 PMC4712381

[B41] Raglio A. Attardo L. Gontero G. Rollino S. Groppo E. Granieri E. (2015). Effects of music and music therapy on mood in neurological patients. *World J. Psychiatry* 5:68. 10.5498/wjp.v5.i1.68 25815256 PMC4369551

[B42] Reber R. Schwarz N. Winkielman P. (2004). Processing fluency and aesthetic pleasure: Is beauty in the perceiver’s processing experience? *Pers. Soc. Psychol. Rev.* 8 364–382. 10.1207/s15327957pspr0804_3 15582859

[B43] Ree A. Denmon C. Tibus E. O. Mayes A. Weatherill M. Auerbach A. (2025). Research on music-based interventions for aphasia: a scoping review. *Aphasiology* 1–28. 10.1080/02687038.2025.2451670

[B44] Rolke B. Stepper M. Y. Seibold V. C. Hein E. (2019). Aesthetic stimuli attract visual spatial attention. *Art. Percept.* 7 52–81. 10.1163/22134913-20191101

[B45] Sacheli L. M. Tomasetig G. Musco M. A. Pizzi S. Bottini G. Pizzamiglio L. (2022). The unexplored link between aesthetic perception and creativity: A theory-driven meta-analysis of fMRI studies in the visual domain. *Neurosci. Biobehav. Rev.* 140:104768. 10.1016/j.neubiorev.2022.104768 35798126

[B46] Sachs M. E. Damasio A. Habibi A. (2015). The pleasures of sad music: A systematic review. *Front. Hum. Neurosci.* 9:404. 10.3389/fnhum.2015.00404 26257625 PMC4513245

[B47] Sachs M. E. Ellis R. J. Schlaug G. Loui P. (2016). Brain connectivity reflects human aesthetic responses to music. *Soc. Cogn. Affect Neurosci.* 6 884–891. 10.1093/scan/nsw009 26966157 PMC4884308

[B48] Sarasso P. Barbieri P. Del Fante E. Bechis L. Neppi-Modona M. Sacco K. (2022a). Preferred music listening is associated with perceptual learning enhancement at the expense of self-focused attention. *Psychon. Bull. Rev.* 29 2108–2121. 10.3758/s13423-022-02127-8 35668293 PMC9722857

[B49] Sarasso P. Neppi-Modona M. Rosaia N. Perna P. Barbieri P. Del Fante E. (2022b). Nice and easy: Mismatch negativity responses reveal a significant correlation between aesthetic appreciation and perceptual learning. *J. Exp. Psychol. Gen.* 151:1433. 10.1037/xge0001149 34793192

[B50] Sarasso P. Perna P. Barbieri P. Neppi-Modona M. Sacco K. Ronga I. (2021). Memorisation and implicit perceptual learning are enhanced for preferred musical intervals and chords. *Psychon. Bull. Rev.* 28 1623–1637. 10.3758/s13423-021-01922-z 33945127 PMC8500890

[B51] Sarasso P. Ronga I. Kobau P. Bosso T. Artusio I. Ricci R. (2020). Beauty in mind: Aesthetic appreciation correlates with perceptual facilitation and attentional amplification. *Neuropsychologia* 136:107282. 10.1016/j.neuropsychologia.2019.107282 31770549

[B52] Sella E. Vincenzi M. Carbone E. Schellenberg E. D. G. Lima C. Toffalini E. (2024). Effects of music listening on cognition and affective state in older adults: A systematic review and meta-analysis. *Eur. Psychol.* 29 199–215. 10.1027/1016-9040/a000533

[B53] Tommasini E. Cipriani E. Antonietti A. Galvani C. (2022). Correlations between physical activity level, quality of life, and cognitive performance in elderly individuals engaging in multi-year dancing activities. *J. Dance Med. Sci.* 26 35–41. 10.12678/1089-313X.031522e 34865684

[B54] Umiltá M. A. Berchio C. Sestito M. Freedberg D. Gallese V. (2012). Abstract art and cortical motor activation: An EEG study. *Front. Hum. Neurosci.* 6:311. 10.3389/fnhum.2012.00311 23162456 PMC3499799

[B55] Valverde-Guijarro E. Alguacil-Diego I. M. Vela-Desojo L. Cano-de-la-Cuerda R. (2022). Effects of contemporary dance and physiotherapy intervention on balance and postural control in Parkinson’s disease. *Disabil. Rehabil.* 44 2632–2639. 10.1080/09638288.2020.1839973 33135935

[B56] Vartanian O. Goel V. (2004). Neuroanatomical correlates of aesthetic preference for paintings. *Neuroreport* 15 893–897. 10.1097/00001756-200404090-00032 15073538

[B57] Vessel E. A. Starr G. G. Rubin N. (2012). The brain on art: Intense aesthetic experience activates the default mode network. *Front. Hum. Neurosci.* 6:66. 10.3389/fnhum.2012.00066 22529785 PMC3330757

[B58] Windle G. Joling K. J. Howson-Griffiths T. Woods B. Jones C. H. Van de Ven P. M. (2018). The impact of a visual arts program on quality of life, communication, and well-being of people living with dementia: A mixed-methods longitudinal investigation. *Int. Psychogeriatr.* 30 409–423. 10.1017/S1041610217002162 29113610

